# Prevalence and Risk Factors of HIV Infection among Clients Attending ICTCs in Six Districts of Tamilnadu, South India

**DOI:** 10.1155/2011/650321

**Published:** 2011-07-19

**Authors:** Rajeswari Ramachandran, V. Chandrasekaran, M. Muniyandi, K. Jaggarajamma, Anasua Bagchi, Supriya Sahu

**Affiliations:** ^1^Tuberculosis Research Centre, Indian Council of Medical Research, Chennai 600 031, India; ^2^Tamilnadu State AIDS Control Society, Health and Family Welfare Department, Chennai 600 008, India

## Abstract

*Objective*. To assess the HIV serostatus of clients attending integrated counseling and testing centres (ICTCs) in Tamilnadu, south India (excluding antenatal women and children), and to study its association with demographic, socioeconomic, and behavioral risk factors. *Design*. In a prospective observational study, we interviewed clients attending 170 ICTCs from six districts of Tamilnadu during 2007 utilizing a standard pretest assessment questionnaire. All the clients were tested for HIV with rapid test kits. Multiple logistic regression analysis was used to identify determinants of HIV infection. *Results*. Of 18329 clients counseled, 17958 (98%) were tested for HIV and 732 (4.1%; range 2.6 to 6.2%) were tested positive for HIV. Median age of clients was 30 years; 89% had never used condoms in their lives and 2% gave history of having received blood transfusion. In multivariate analysis HIV seropositivity was associated with HIV in the family (adjusted odds ratio) (AOR 11.6), history of having sex with sex workers (AOR 2.9), age ≥31 years (AOR 2.8); being married (AOR 2.5), previously tested for HIV (AOR 1.9), illiteracy (AOR 1.7), unemployment (AOR 1.5), and alcoholism (AOR 1.5). *Conclusion*. HIV seroprevalence being high in ICTC clients (varied from 2.6 to 6.2%), this group should also be included in routine programme monitoring of sero-positivity and risk factors for better understanding of the impact of the National AIDS Control Programme. This would help in evolving appropriate policies and strategies to reduce the spread of HIV infection.

## 1. Background

India today stands at the crossroads in its battle against Human Immunodeficiency Virus/Acquired Immune Deficiency Syndrome (HIV/AIDS). Responding to the immense challenge of the HIV/AIDS threat, National AIDS Control Programme (NACP) has articulated a clear and effective response to increase access to services and communicate effectively for behaviour change. The overall goal of NACP is to halt and reverse the epidemic in India over the next five years by integrating programmes for prevention, care and support, and treatment. In Tamilnadu, Voluntary Counselling and Testing Facilities (VCTFs) were established in healthcare facilities to promote access to HIV counselling and testing from the year 2004. Voluntary counselling and testing services provide an entry point for prevention and care. The concept of integrating the preventive and care services was initiated through the initiation of Integrated Counselling and Testing Centres (ICTCs) in Tamilnadu from 2005. The ICTCs provide pretest counseling, testing, retesting, posttest counseling, and support services. Clients (including general population, high risk groups, and referrals) utilize the services of these centres on voluntary basis. 

**Figure 1:**
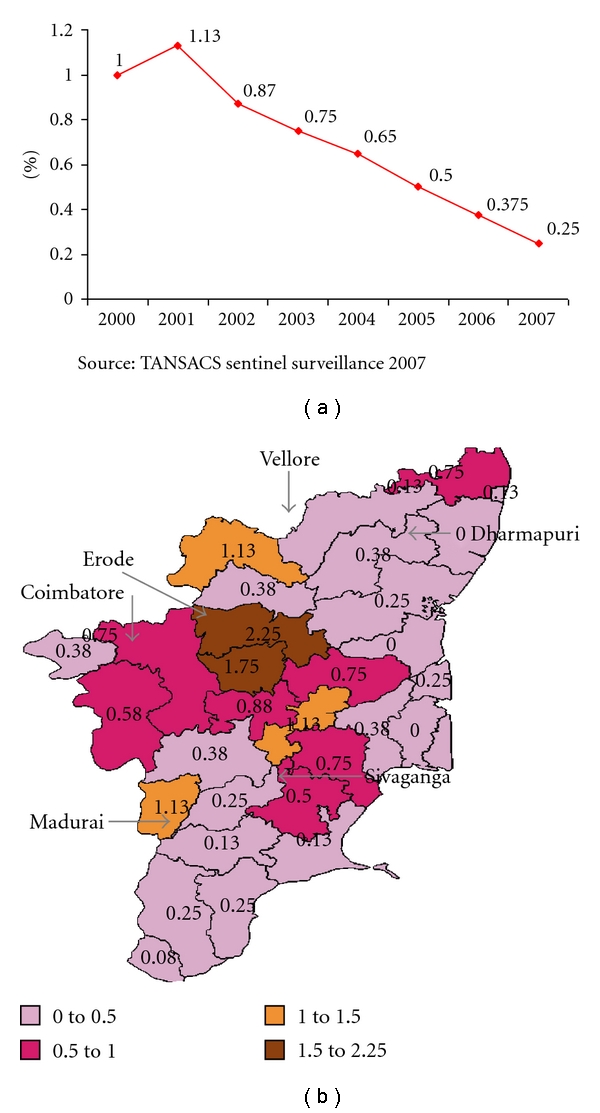
HIV prevalence among Ante Natal cases in Tamilnadu.

Tamilnadu has been one of the 6 states in India with high prevalence of HIV among the antenatal care (ANC) cases (>I%, Figure 1). The major drivers of the epidemic in the state are highways connecting neighboring high prevalent states, multiple truckers halt points with hub of commercial sexual activities, industrial belts encouraging in migration from multiple states, and traditional sex trade in some districts. Tamilnadu AIDS Control Society (TANSACS), first state AIDS cell established in India that was set up in 1989, has come a long way in its effort to control the HIV epidemic in the state. Strong political will and bureaucratic commitment to halt and reverse the epidemic with continued evidence-based strategic planning has established Tamilnadu as a trend-setter in combating HIV AIDS in India and in achieving the goals of National AIDS Control Program Phase III. It is estimated that there are 0.14 million people living with HIV in the state. With continued focused Prevention and Care Program in the state, Tamilnadu has achieved a decline in the HIV prevalence in the state to 0.25% of ANC, which also contributes to the apparent reduction in HIV prevalence in south India as a whole [1]. The decline in HIV prevalence in Tamilnadu (John, 2006) [2] is supported by the program data as well HSS conducted over the years. 

Information on HIV prevalence among patients attending STD clinics, commercial sex workers, MSM, and also among general population is available in India [3–5]. However, there are very few reports about the seroprevalence of HIV among clients attending the ICTCs [6]. The objective of this present study is to assess the rate of HIV seropositivity and risk factors of HIV infection among these clients attending ICTCs, excluding antenatal women and children, in six districts of Tamilnadu, south India.

## 2. Methodology

### 2.1. Setting

As of December 2007, a total of 760 ICTCs were operational in Tamilnadu state with trained counselors, laboratory technicians, and outreach workers. All the ICTC counsellors were trained for counselling the clients for HIV screening and filling-up standard pretest assessment forms. After proper counseling and getting written consent blood test was done to confirm HIV serostatus. Three rapid HIV test kits (COMB. AID HIV 1/2, HIV TRI DOT, and HIV COMB) were used for the purpose of diagnosis of HIV, every positive result was reconfirmed by using two more rapid test kits before giving final result to the person. 

All ICTCs participate in an external quality assessment scheme (EQAS). Each ICTC is assigned a “State Reference Laboratory” (SRL). EQAS involves sending of “coded” samples from the reference laboratories to the ICTCs twice a year for testing. In addition, ICTCs send 20% of all positive samples and 5% of all negative samples collected in the first week of every quarter for cross-checking to the SRL once every quarter.

### 2.2. Study Area

**Figure 2:**
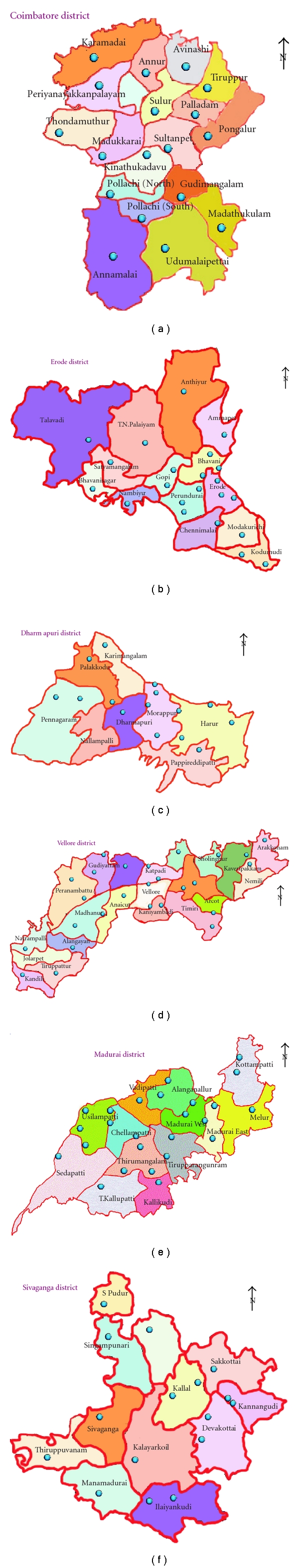
Distribution of ICTCs in the Districts—Coimbatore, Erode, Vellore, Dharmapuri, Madurai, and Sivaganga.

This study was carried out in six selected districts of Tamilnadu state, namely, Coimbatore, Erode, Vellore, Dharmapuri, Madurai, and Sivaganga (based on the clients attended). These districts covered a total population of 17.1 million (ranging from 1.2 to 4.3), and total number of ICTCs functioning were 170 (ranging from 14 to 45 per district; Figure 2).

### 2.3. Study Population

All clients attending ICTCs for HIV screening on a voluntary basis or referred basis for a period of one month during May to December 2007 formed the study population (each district approximately 4–6 weeks), excluding pregnant women and children. Study procedures were approved by Institutional Ethics Committee of Tuberculosis Research Centre (TRC).

### 2.4. Tools Used for Data Collection

Semistructured and precoded standardized National AIDS Control Organization (NACO) approved “Pretest Assessment Form” implemented by Tamilnadu AIDS Control Society (TANSACS) was used for the study.

### 2.5. Information Collected

All clients were informed in their local language about the purpose of the study and written consent was obtained before proceeding to the interviews. The interviewer told about the confidentiality of data and the data management at TRC. 

The interview schedule included demographic, socio-economic characteristics (education, occupation), history of risk behaviour, history of HIV in the family, and life style indicators like smoking and alcoholism. We also collected the information on HIV status of the clients screened from the ICTCs records.

### 2.6. Duration of the Study

This study was carried out between January and December 2007, and for each district data for 4–6 weeks was collected.

### 2.7. Data Management

Data were collected by trained field investigators and scrutinized by the investigators, and random checks were done to ensure quality of data. Data was entered in DataStar and analyzed using the SPSS package.

### 2.8. Data Analysis

An analysis of the frequency distribution of patients profile with respect to demographic, socio-economic status was made. In univariate analysis, the Chi-square test was used to compare the HIV seropositivity rate in different demographic socioeconomic groups of clients attending ICTCs. Adjusted odd ratios and 95% confidence intervals were reported as measures of effect size for the relationship between each dichotomous variable and HIV status. A *P*-value <0.05 was considered statistically significant. Following these univariate tests, all selected variables were entered into a logistic regression analysis with HIV status as the outcome variable using SPSS/PC, to find out the factors independently associated with HIV seropositivity (adjusting for the confounding factors). The outcomes of both significant and nonsignificant variables are reported.

## 3. Results

### 3.1. District Profile

Table 1 describes the population of districts under the study, number of ICTCs available, number of clients screened (excluding antenatal women and children), and the seroprevalence. A total of 18329 clients were counseled by ICTC counselors during 2007 from six districts of Tamilnadu state, and among them for 17958 (98%) HIV serology was available and the overall HIV seroprevalence was 4.1% (varied from 2.6 to 6.2% in different districts).

### 3.2. Demographic and Socioeconomic Profile

Of those who were screened 11725 (65.5%) were males, 6159 (34.4%) were females, and 18 (0.1%) were transgender. Of the clients screened 12468 (69%) were screened at tertiary care centers like medical colleges and district head quarter hospitals, and the remaining clients were screened at primary care centres. The median age of the clients was 30 years; 27% were illiterates, 17% were unemployed, 9% were unmarried, and 97% were living with their families.

### 3.3. Characteristics of Clients

Eighty nine percent (87% males; 91% females) had never used condoms in their lives. Only 2% of the clients gave a history of having received blood transfusion, 7% having had sex with sex workers, and 11% having had sex with own sex. There was history of HIV in the family in 4%, and 7% had been tested for HIV earlier. History of alcoholism and injecting drug was observed in 37% and <1%.

### 3.4. Risk Factor for HIV Infection

Table 2 describes the estimated HIV seropositivity rate among different demographic, socio-economic groups and clients with risk behaviours attending ICTCs for HIV screening. The seroprevalence was similar among males (3.9%) and females (4.4%). The seroprevalence observed among clients attending the tertiary care centres was significantly higher (4.7% versus 2.6%; *P* < 0.01) as compared to clients from primary care centres. Univariate analysis demonstrated that a higher HIV seroprevalence was significantly associated with clients aged ≥31 years, illiterates, unemployed, widower/divorced, living with outside family, history of having sex with sex workers, HIV in the family, previously tested for HIV, history of alcoholism and drug addiction. After adjusting for confounding factors by logistic regression analysis the higher seroprevalence was observed among the clients with HIV in the family adjusted odds ratio (AOR): 11.6; 95% CI:8.0–16.9), history of having sex with sex workers (AOR: 2.9; 95% CI: 2.0–4.3), aged ≥31 years (AOR: 2.8; 95% CI: 2.0–4.0), married (AOR: 2.5; 95% CI: 1.5–4.1), previously tested for HIV (AOR: 1.9; 95% CI: 1.3–2.8), illiterates (AOR: 1.7; 95% CI: 1.3–2.4), unemployed (AOR: 1.5; 95% CI: 1.1–2.1), and alcoholism (AOR: 1.5; 95% CI: 1.1–2.1).

When all the risk factors were compared among males and females, except for age there was no significant difference between the sexes. The risk was higher among females ≤30 years (OR 2.15; 95% CI: 1.64–2.82; *P* < 0.001).

## 4. Discussion

The present study has documented the prevalence and risk factors of HIV infection among a large number (*N* = 17958) of clients attending ICTCs (excluding antenatal women, since surveillance in this group is being carried out routinely) situated in six districts of Tamilnadu, south India. The present study highlights 4.1% HIV seropositivity among the clients utilizing the ICTCs. This was high as compared to seroprevalence among the general population (0.34% from community based household survey [7], 0.36% based on national sentinel surveillance system [8], and 0.5% among antenatal women in Tamilnadu) [9], but similar to HIV seroprevalence among patients attending STD clinics (3.74%), female sex workers (4.9%) [6], and TB patients (4.7%) [10]. Our study findings show high HIV infection among the clients aged ≥31 years, males, illiterates, unemployed, widower/divorced, living and outside family, with history of having sex with sex workers, HIV, in the family, previously tested for HIV, and alcoholics. To our knowledge this is the first report on the seropositivity and the risk factors among clients attending ICTCs. 

In the present study 89% had never used condoms in their lives. Also one third of the study population gave premarital sexual experience. Studies conducted in India and Vietnam report similar observation on premarital sex [11, 12]. The young adults who migrate on account of work and stay away from the family are at risk to acquire HIV infection because they are known to buy sex and do not have access to information, condoms, or supportive services that would enable them to have safe sex [13, 14]. This finding suggests the need for the scaling-up of focused prevention efforts among these groups.

The noteworthy finding observed in this study was that the illiterates and unemployed were at higher risk. This observation corroborates with the finding of the Third National Family Health Survey where HIV seroprevalence was higher among population living with the lowest wealth as compared to those living with the highest wealth (0.27% versus 0.18%) [6]. The poor are characterized by weak endowments of human (education) and financial resources, few marketable skills, and generally poor health all of which result in low productivity. These characteristics increase the risk of infection and inadequate access to health facilities in poor communities, leading to higher chance to facilitate the HIV transmission. Therefore HIV is more prevalent in poor socioeconomic communities [15, 16]. This major factor may be useful for health planners and policy makers for better allocation of resources in controlling the spread of HIV infection. 

In India, the majority of the population is still not infected with HIV. Prevention strategies must continue to be given primary focus through awareness campaigns, usage of condoms, and counseling facilities, which will lead to behavioral change. With the increase in awareness levels in the community, the demand for voluntary counseling and testing services would rise. Specific groups like illiterates, unemployed, sex workers, and alcoholics need specially packaged awareness programmes on the risk and vulnerability to HIV/AIDS.

## 5. Limitation of Study

All clients interviewed attended the ICTCs for HIV screening on a voluntary basis or referred basis. The interview schedule included sensitive data like the history of risk behavior and history of HIV in the family, and it is possible that the data reported here could be biased due to recall error and social stigma faced by the patients in the community. In addition, 2% of our clients were not screened for HIV. However this small proportion of sample may not have influenced the outcome of this study finding.

## 6. Conclusion

ICTCs are important for prevention, detection, and care of HIV infection. This study reports for the first time the seroprevalence of HIV among clients attending ICTC (excluding antenatal women and children) in Tamilnadu. HIV seroprevalence being high in this group, this group should also be included in routine programme monitoring of seropositivity and risk factors. This could be utilized for assessing the impact of HIIV/AIDS control measures. This would help in the development of the appropriate policies and strategies to reduce the spread of HIV infection.

## Figures and Tables

**Table 1:** Study participants from different ICTCs in six districts of Tamilnadu and HIV seropositivity (excluding antenatal women and children).

Name of the districts	Populationin million	No. ICTCs	No. counselors trained	No. clients screened	HIV seropositivity
Madurai	2.6	25	24	4473	193 (4.3%)
Sivaganga	1.2	19	26	1694	47 (2.8%)
Coimbatore	4.3	37	38	3703	145 (3.9%)
Erode	2.6	30	29	3169	81 (2.6%)
Dharmapuri	2.9	14	13	1808	73 (4.0%)
Vellore	3.5	45	33	3111	193 (6.2%)

Total	17.1	170	163	17958	732 (4.1%)

**Table 2:** HIV prevalence and determinants among clients attending ICTCs in six districts of Tamilnadu (*N* = 17958).

Characteristic	Positive (N)	Negative (N)	Positive %	Missing %	Crud OR (95% CI)	Adjusted OR (95% CI)
Age (Years)				1.5		
≤30	227	9172	2.4		2.6 (2.2–3.0)**	2.8 (2.0–4.0)**
≥31	500	7791	6.0			
Sex				0.3		
Male	459	11266	3.9		1.1 (1.0–1.3)	—
Female	271	5906	4.4			
Education				3.3		
Illiterate	243	4443	5.2		1.5 (1.3–1.8)**	1.7 (1.3–2.4)**
Literate	449	12224	3.5			
Occupation				8.9		
Unemployed	171	2977	5.4		1.4 (1.2–1.7)**	—
Employed	502	12705	3.8			
Marital				3.0		
Unmarried	63	1428	4.2			—
Married	580	14893	3.7		—**	2.5 (1.5–4.1)**
Widower/Divorced	71	379	15.8			1.2 (0.5–2.5)
Living with				5.2		
Others	40	499	7.4		2.0 (1.4–2.7)**	—
Family	652	15827	4			
Received blood				4.0		
Yes	34	316	9.7		2.7 (1.8–3.9)**	—
No	653	16231	3.9			
Premarital sex				6.0		
Yes	255	5535	4.4		1.1 (1.0–1.3)	—
No	432	10660	3.9			
Sex with a sex worker						
Yes	143	1081	11.7	7.5	3.6 (3.0–4.4)**	2.9 (2.0–4.3)**
No	539	14856	3.5			
Use condom in their life				5.8		
Yes	104	1835	5.4		1.4 (1.1–1.7)*	—
No	586	14389	3.9			
Centre				0		
Tertiary	592	11876	4.7		1.9 (1.6–2.3)**	—
Nontertiary	140	5350	2.6			
Sex with a person of own sex				10.0		
Yes	64	1641	3.8		0.9 (0.7–1.2)	—
No	578	13869	4			
HIV in the family				14.6		
Yes	167	493	25.3		11.4 (9.2–14.0)**	11.6 (8.0–16.9)**
No	425	14258	2.9			
Previous HIV test				9.6		
Done	151	978	13.4		4.5 (3.6–5.4)**	1.9 (1.3–2.8)**
Not done	505	14605	3.3			
Alcoholism				37.0		
Yes	201	3953	4.8		1.6 (1.3–1.9)**	1.5 (1.1–2.1)**
No	225	6929	3.1			
Injecting drugs				17.5		
Yes	8	50	13.8		4.1 (1.8–9.1)**	—
No	551	14213	3.7			

*P < 0.05; **P < 0.01; OR: Odds Ratio; CI: Confidence Interval.
